# Malignant Pleural Effusion Due to Uterine Serous Carcinoma: An Unusual Presentation

**DOI:** 10.7759/cureus.34354

**Published:** 2023-01-29

**Authors:** Mustafa Wasifuddin, Nosakhare Ilerhunmwuwa, Ifeanyi Uche, Henry O Aiwuyo, Narek Hakobyan, Ephrem Sedeta, Jamal C Perry, Beatrice E Torere, Hesham Ali Abowali, Larisa Mararenko

**Affiliations:** 1 Internal Medicine, Brookdale University Hospital Medical Center, Brooklyn, USA; 2 Internal Medicine, North Mississippi Medical Center, Tupelo, USA; 3 Hematology and Oncology, Brookdale University Hospital Medical Center, Brooklyn, USA

**Keywords:** endometrial carcinoma, rare cause of pleural effusion, serous endometrial carcinoma, malignant pleural effusion, uterine cancer

## Abstract

Endometrial cancer is the most common cancer of the female genital tract. It can rarely metastasize to the pleura and present as a malignant pleural effusion. Here we present the case of a 61-year-old female with two primary malignancies, breast and endometrium, who presented to us with shortness of breath. Imaging was suggestive of a malignant pleural effusion. Diagnostic and therapeutic thoracentesis were performed that were initially suggestive of a breast source. However, final pleural fluid studies showed endometrial serous carcinoma as the source of the effusion. The patient received pembrolizumab and lenvatinib treatment and continues to be followed up in our clinic.

## Introduction

Endometrial carcinoma rarely metastasizes to the lungs despite being the most common cancer of the female genital tract. It has been estimated that approximately 4% of patients with endometrial carcinoma develop lung metastases - a sign of disseminated disease. When the disease spreads to the lungs, solitary or multiple pulmonary nodules are common. However pleural metastases are fairly uncommon, and solitary pleural metastases without lung parenchymal involvement are rare. These can rarely present as malignant pleural effusions in metastatic endometrial carcinoma [1]. Extrauterine involvement most commonly results in lymph nodal disease of the paraaortic and pelvic lymph nodes [2]. Other extrauterine sites of spread include peritoneum, brain, bones and liver [3]. We present a patient who was diagnosed with endometrial serous carcinoma after presenting with a malignant pleural effusion.

## Case presentation

**Figure 1:**
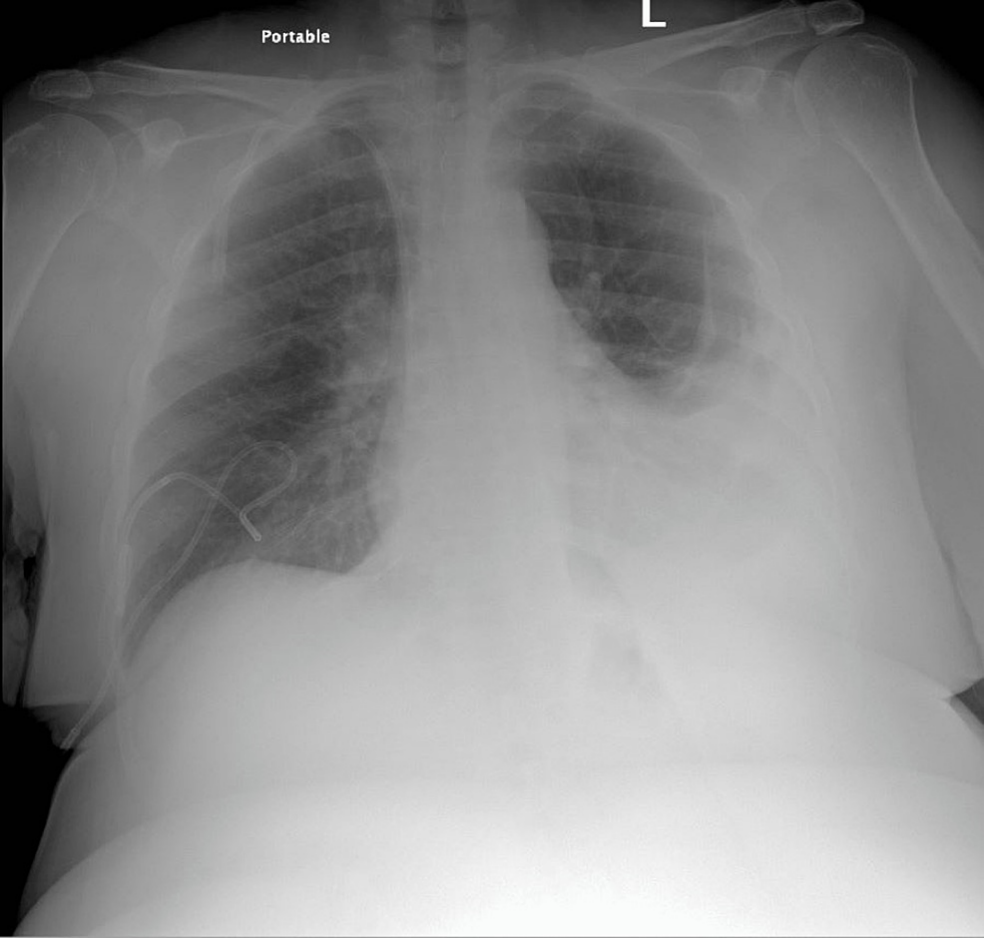
Chest x-ray of our patient demonstrating a left sided pleural effusion

A 61-year-old female presented with two months of worsening shortness of breath with associated dry cough. She had medical history significant for diabetes mellitus, hypertension, estrogen receptor negative (ER-), progesterone receptor negative (PR-) and human epidermal growth factor receptor 2/neu negative (HER2/neu3+) infiltrative ductal carcinoma of the right breast that was previously treated with chemotherapy and total mastectomy. She was found to be hypoxic with an oxygen saturation of 92%. Chest x-ray showed left mid to lower lung field opacification (Figure 1).

**Figure 2:**
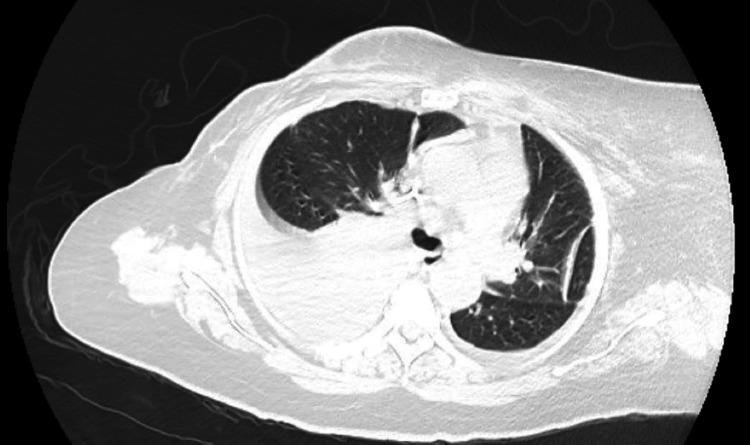
Chest CT of our patient demonstrating a left sided pleural effusion

**Figure 3:**
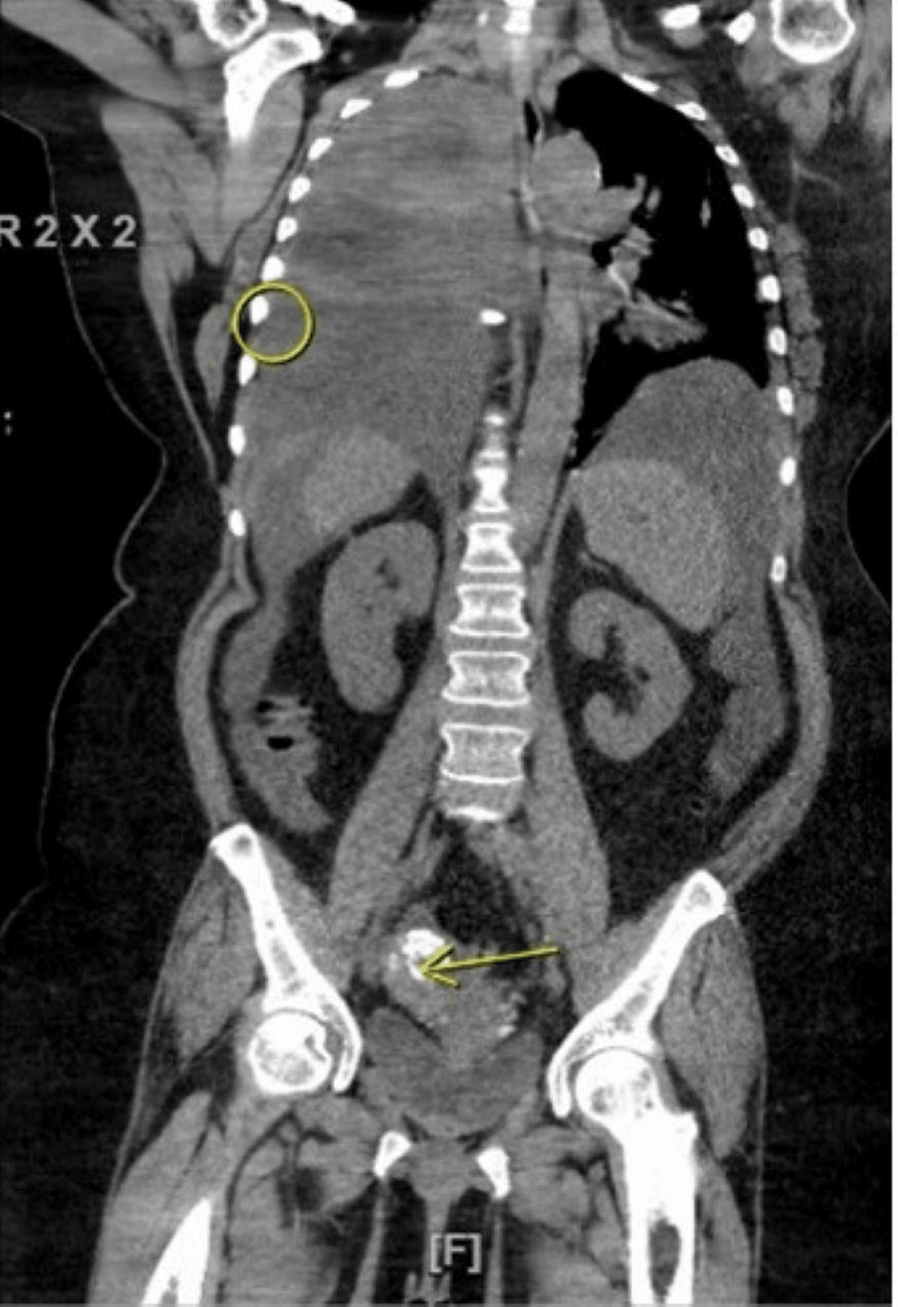
Circled area showing pleural effusion and arrowed area showing calcified endometrial tumor

A CT angiography of the chest showed a moderate to large left-sided pleural effusion with moderate left lower lung compressive atelectasis (Figure 2). Ultrasound-guided left thoracentesis was performed and approximately 1.2 L of hemorrhagic fluid was aspirated. Pleural fluid was sent for analysis. The patient had immediate symptomatic relief. However, she became short of breath and hypoxic again three days later. Repeat chest x-ray showed reaccumulation of fluid in the pleural space. A repeat thoracentesis was performed with removal of 200mL of pleural fluid. A pigtail chest tube was placed that improved symptoms. A CT scan of the chest, abdomen and pelvis showed thickening of the anterior peritoneum, stranding and nodularity of the adjacent mesentery with moderate amount of perisplenic and perihepatic ascites. This was concerning for metastatic disease. There was no evidence of lung parenchymal involvement (Figure 3).

**Figure 4:**
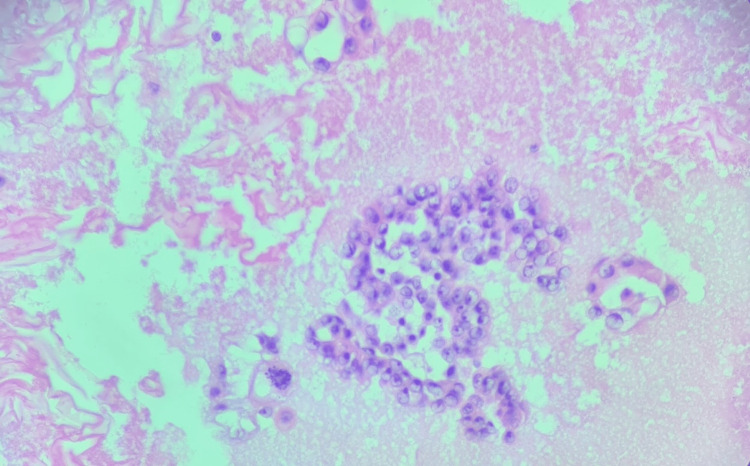
H&E stain of pleural fluid showing large nucleoli, mitotic figures and glands forming high grade adenocarcinoma

**Figure 5:**
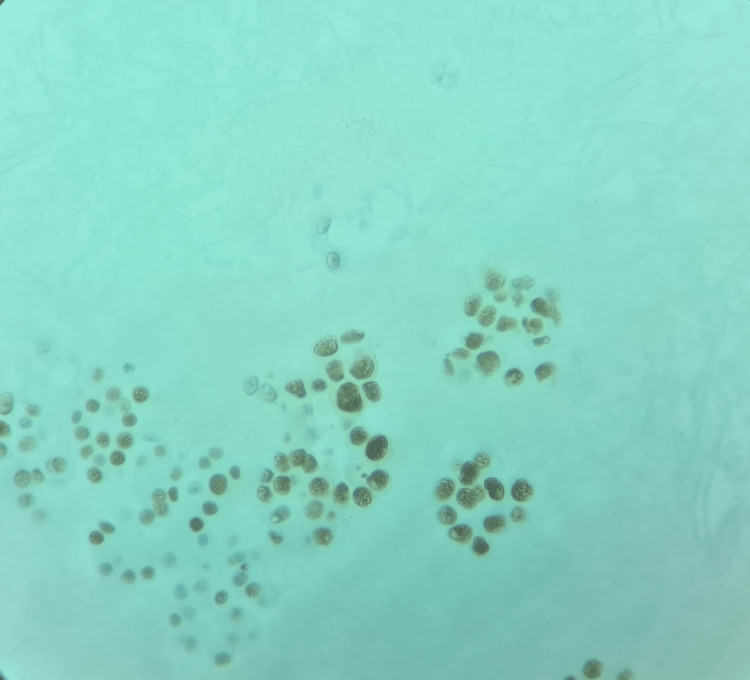
P53 stain of sample showing many tumor clusters with large nuclei

**Figure 6:**
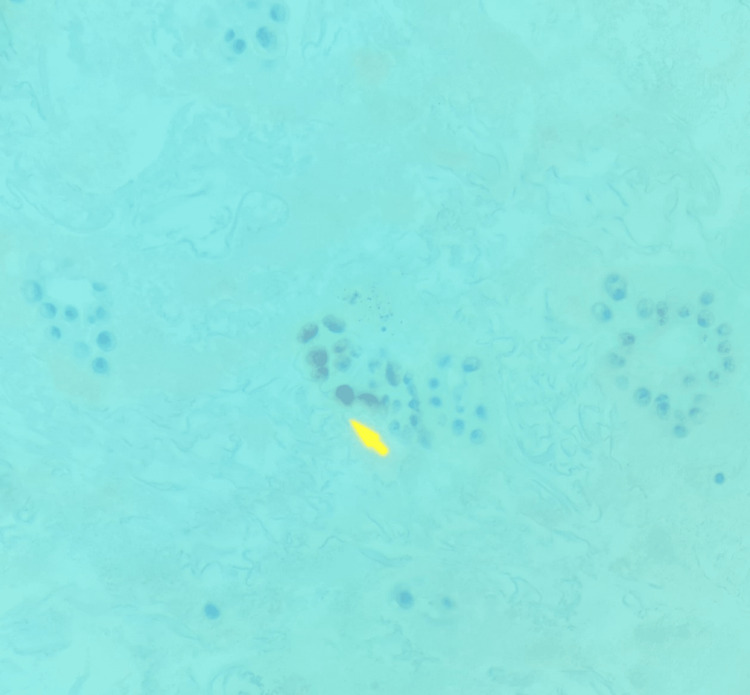
Immunohistochemistry staining of pleural fluid sample for PAX-8 shows positive staining for endometrial cancer, serous subtype

Bone scan was positive for degenerative changes. Preliminary pleural fluid analysis from the initial aspirate showed an exudative pattern with malignant cells showing adenocarcinoma of breast origin. Immunostaining was requested for the pleural fluid. The patient was offered pleurodesis due to continued pleural fluid drainage but she declined the intervention. A PET scan was suggestive of hypermetabolic activity in the uterine cavity, thoracic, mediastinal and internal mammary lymph nodes. Omental and peritoneal nodularity was noted. Hypermetabolic pleural effusion and ascites were also noted. Omental biopsy was done and showed features suggestive of papillary and psammoma bodies. Ascitic fluid study was suggestive of high-grade adenocarcinoma with immunohistochemistry suggestive of adenocarcinoma of mullerian origin. An endometrial biopsy was performed that was suggestive of serous endometrial carcinoma. Final pleural fluid analysis showed tumor cells (Figure 4) positive for uterine serous primary carcinoma markers p53 (Figure 5), PAX8 (Figure 6), and P16 and negative for breast markers GATA3, GCDFP15, and mammaglobin, thereby favoring metastatic uterine papillary serous carcinoma. Surgery was ruled out to due to the advanced stage and progressive nature of the uterine cancer. The patient was scheduled for chemotherapy with carboplatin, paclitaxel and avastin. She received five cycles of this chemotherapy regimen before she developed peripheral neuropathy. She was subsequently switched to pembrolizumab and lenvatinib. The pleural catheter was then removed and she was discharged to follow up in our outpatient hematology/oncology clinic.

## Discussion

Endometrial cancer is the most commonly diagnosed cancer of the female genital tract [4-6]. The majority of endometrial cancers have a favorable prognosis as they are localized to the uterus upon presentation and are of the endometrioid histological subtype, accounting for nearly 75-80% of cases [3,4]. The more aggressive subtypes have an increased risk of metastatic disease and include mixed, squamous cell, clear cell and serous carcinoma [3]. They are responsible for the majority of the deaths in patients with endometrial carcinoma. One of the aggressive subtypes, uterine serous carcinoma, accounts for 10% of the cases of endometrial cancer. However, it is responsible for nearly 40% of endometrial cancer related deaths [4,5]. Our patient is of the serous carcinoma subtype. The higher proportion of deaths from the serous subtype can be partially attributed to the higher stage at which endometrial serous carcinoma patients present. The International Federation of Gynecology and Obstetrics for the 1999-2001 period reported 37% of endometrial serous cancers displaying no invasion in the uterus were found to have stage III or IV disease [3]. 

The overall incidence of disseminated disease for endometrial cancer is 2-4% [3]. However, arising primarily in post-menopausal women, serous carcinoma of the uterus is very aggressive and has a tendency for early hematogenous and lymphatic spread. It also has a tendency for intraperitoneal spread [2]. Lung is one of the organs to which endometrial cancer may spread. However, pleural spread is very uncommon in this disease. A study conducted in India found the incidence of pleural effusion among endometrial cancers to be between 6-10% [7,8]. There are isolated case reports in the literature whereby uterine serous carcinoma presents with pleural effusion in the recurrent setting or in the already diagnosed patient [1,2]. There has not been, to our knowledge, previous reports of malignant pleural effusion as the diagnostic presentation of serous endometrial carcinoma in a patient with history of breast carcinoma. Our case is unique in that the diagnosis of serous endometrial cancer was made after she presented with a malignant pleural effusion. In cases where two primary cancers exist in a patient, like in ours, it can be particularly difficult to identify the source of the malignant pleural effusion. Initial review of our patient's pleural fluid aspirate demonstrated adenocarcinoma cells. Based on our patient’s history of recurrent breast cancer and these pleural fluid analysis results, breast cancer was initially identified as the source. In fact, a treatment plan had been formulated to treat our patient as a breast cancer recurrence. However, detailed pleural fluid analysis with immunostaining showed cells of mullerian origin. It was positive for p53 (Figure 4), PAX8 (Figure 5), and P16 and negative for breast markers GATA3, GCDFP15, and mammaglobin, thereby favoring metastatic uterine papillary serous carcinoma as the source. It prompted us to change our treatment course to the correct chemotherapy regimen. Therefore, a decision on the source of malignant pleural effusion must be made only after complete analysis of fluid cytology and immunohistochemistry to select the correct treatment regimen for patients.

Treatment for metastatic endometrial carcinoma involves cytoreduction with surgery and treatment with chemotherapy [2,9]. Omentectomy is recommended in patients with serous, carcinomasarcoma and undifferentiated endometrial carcinoma. Patients who undergo surgery should receive adjuvant chemotherapy with carboplatin and paclitaxel. Pelvic radiation therapy may also be added as an adjuvant after surgery in combination with chemotherapy [10]. According to the American Society of Clinical Oncology, pembrolizumab and chemotherapy with or without bevacizumab may be offered to patients with persistent, recurrent or metastatic endometrial carcinoma [11]. Pembrolizumab is an antibody that binds to PDL-1 on lymphocytes and potentiates the cell mediated response. Bevacizumab is a humanized monoclonal antibody that inhibits VEGF-A, thereby inhibiting angiogenesis. Hormone therapy is a viable alternative in patients with estrogen receptor positivity who have progression of disease. Treatment of malignant pleural effusions secondary to endometrial cancer include therapeutic thoracentesis, pleurodesis and in refractory cases pleurectomy [2,12,13].

## Conclusions

Malignant pleural effusion secondary to metastatic serous carcinoma of the uterus is an extremely rare clinical phenomenon. It is important to operate with vigilance in patients with a known history of malignancy presenting with malignant pleural effusion to correctly identify the cause to allow accurate and timely management. Pleural fluid cytology and immunohistochemistry are powerful tools that must be used whenever possible in patients presenting with a malignant pleural effusion.
